# Crushing Consequences: A Case Report on the Rehabilitation of a Middle Phalangeal Fracture in an Industrial Incident

**DOI:** 10.7759/cureus.63965

**Published:** 2024-07-06

**Authors:** Chaitali S Vikhe, Swapnil U Ramteke, Pratik R Jaiswal

**Affiliations:** 1 Sports Physiotherapy, Ravi Nair Physiotherapy College, Datta Meghe Institute of Higher Education & Research, Wardha, IND

**Keywords:** post-operative management, mulligan mobilisation with movement, multimodal rehabilitation, k wire fixation, middle phalanx fracture

## Abstract

Hand injuries, particularly fractures involving the phalanges and metacarpals, are common occurrences in various settings, including industrial environments. Prompt and effective management of these injuries is crucial to minimize long-term disability and facilitate return to work. This case report focuses on the rehabilitation of a middle phalangeal fracture in an industrial worker following Kirschner wire (K-wire) fixation. The patient, a 24-year-old male, sustained the injury while operating an electric stamping machine. Emergency surgery was performed to stabilize the fracture, and subsequent physiotherapy was initiated due to persistent difficulties in hand function. The rehabilitation protocol aimed to address pain, improve range of motion, and enhance grip strength through passive range of motion exercises, movement with mobilization techniques, blocking exercises, and grip strengthening exercises. Mulligan's mobilization with movement (MWM) concept was incorporated to facilitate rapid pain relief and movement restoration. Follow-up assessments after four weeks of rehabilitation revealed significant improvements in pain levels, range of motion, strength, and overall quality of life. The case underscores the importance of timely intervention and comprehensive rehabilitation strategies in managing traumatic hand injuries in industrial settings, aiming to optimize treatment outcomes and promote successful return to work.

## Introduction

Hand injuries, which are often underestimated and overshadowed by long bone fractures, demand meticulous attention due to their intricate nature. The management of hand fractures can be a delicate balance, with complications ranging from deformity due to undertreatment, stiffness from overtreatment, to a combination of both from poor treatment. The human hand, being the most vulnerable part of the body, is susceptible to injuries across diverse settings, including industrial, sports, agriculture, and accidents. Among hand fractures, those involving the phalanges and metacarpals constitute a significant portion, accounting for 10% of upper extremity fracture cases. Phalangeal fractures, in particular, can have a profound impact on hand function [1]. Kirschner wire (K-wire) fixation is a technically easy and universally available method, offering flexibility with single or multiple wires and crossed configurations [2].

Despite its advantages in expeditiousness, low cost, and removability, it comes with challenges, including reduced stability and potential postoperative complications such as pin site infections, fixation loss, and delays in rehabilitation [3]. Interphalangeal fractures, especially when unstable or open, often necessitate surgical intervention. Percutaneous K-wire fixation is the least invasive method among the various fixation options, potentially minimizing the risks of tendon adhesions and joint stiffness. However, the choice of treatment method plays a crucial role in determining the outcome, with varying complication rates across injuries and techniques [4]. Postoperative rehabilitation is a critical aspect of managing proximal phalangeal fractures, aiming to optimize hand function. A disparity in clinical practice exists concerning the early controlled mobilization of extraarticular fractures fixed with K-wires. While conventional wisdom delays mobilization for three weeks, the rationale behind this practice remains unclear. An alternate perspective suggests that early controlled mobilization may mitigate stiffness, muscle weakness, and adhesions associated with immobilization, potentially improving fracture healing [5]. Workplace injuries, particularly those affecting the hands, are alarmingly common, often involving a young and productive population. Machinery plays a significant role in these injuries, emphasizing the need for enhanced workplace safety measures [6].

Proprioceptive neuromuscular facilitation (PNF) was introduced in the 1950s, initially termed proprioceptive facilitation. The concept of PNF was originally developed for rehabilitation purposes. This work was supported by the Kaiser Institute in the 1940s. The International PNF Association (IPNFA) identifies itself as the official successor of the original developers [7]. PNF, a cornerstone of rehabilitation, is extensively employed by physiotherapists and integrated into physiotherapy education across various nations. Regarded as a holistic rehabilitation method, PNF fosters motor learning, control, strength, and mobility. This encompassing approach to rehabilitation entails task-specific training coupled with manual facilitation to enhance motor learning and control [8]. Blocking exercises typically occur once the fracture is clinically healed. These exercises aim to enhance joint movement, prevent stiffness, and improve overall functionality [9]. Mulligan's theory from 1999 suggests that the success of mobilization with movement (MWM) in providing rapid pain relief and restoring movement is attributed to its ability to correct positional faults of the bony segments [10]. MWM entails applying manual force to facilitate translational or rotational articular glides, aiming to stimulate active physiological movement [11]. This emphasizes the importance of precise and controlled exercises in the rehabilitation protocol, contributing to a more effective recovery process for individuals with phalanx fractures [12]. The aim of this study is to prevent complications, enhance treatment outcomes, and contribute insights that could improve management and back to work in the industrial setting.

## Case presentation

A 24-year-old male industrial worker had an accident while working with an electric stamping machine. The machine unexpectedly activated, and his left hand got trapped inside the machine; then he removed his left hand from the machine on his own and informed his head, they wrapped the hand in cloth, and he was quickly taken to a local hospital, where the severity of the injury initially raised concerns about hand amputation. Seeking a second opinion, the patient was referred to a super specialty hospital. X-rays revealed a middle phalangeal fracture of the middle finger of the left hand. For this, emergency surgery was planned where K-wire fixation was performed to stabilize the fracture. The K-wire was removed after seven days, and after one month, the patient was referred for physiotherapy due to persistent difficulties. At the time of physiotherapy referral, the patient complained of difficulty making fists, completely extending fingers, and challenges in performing activities of daily living (ADL). This case highlights the rapid response to a traumatic hand injury in an industrial setting, emphasizing the importance of timely intervention and ongoing rehabilitation.

Clinical findings

On examination, the patient was conscious, cooperative, and oriented to time, place, and person. It was noted that the patient was wearing a dorsal blocking splint, and all fingers of the left hand were positioned in a flexed position within the splint; the patient experienced severe pain with all movement and stiffness in that hand. Additionally, there was a decrease in strength, limited range of motion, and compromised grip. The use of a splint and the observed symptoms highlight the need for careful management to address pain and improve left-hand mobility during the rehabilitation. The patient reported pain levels using the Numerical Pain Rating Scale (NPRS) is a commonly used method to quantify pain intensity on a scale from 0 to 10, where 0 represents no pain and 10 represents the worst pain imaginable. The pain levels are reported for two conditions: pain on movement and pain at rest. The patient reported an 8 out of 10 pain level when moving the affected hand. The patient reported a 4 out of 10 pain level when the affected hand was at rest. Table 1 illustrates the pre-intervention findings of the range of motion, indicating a reduction in flexibility across the metacarpophalangeal (MCP), proximal interphalangeal (PIP), and distal interphalangeal (DIP) joints.

**Table 1:** Pre-intervention range of motion of affected hand (left hand) MCP - metacarpophalangeal joint; PIP - proximal Interphalangeal joint; DIP - distal Interphalangeal joint; NA - not assessed

Movements		Pre-rehabilitation				
		Thumb	Index finger	Middle finger	Ring finger	Little finger
MCP	Flexion	0⁰-45⁰	65⁰-70⁰	55⁰-60⁰	65⁰-70⁰	60⁰-70⁰
	Extension	45⁰-0⁰	70⁰-65⁰	60⁰-55⁰	70⁰-65⁰	70⁰-60⁰
PIP	Flexion	0⁰-80⁰	40⁰-45⁰	30⁰-35⁰	40⁰-45⁰	40⁰-50⁰
	Extension	80⁰-0⁰	60⁰-55⁰	35⁰-30⁰	45⁰-40⁰	50⁰-40⁰
DIP	Flexion	NA	20⁰-25⁰	20⁰-22⁰	20⁰-25⁰	15⁰-25⁰
	Extension	NA	25⁰-20⁰	22⁰-20⁰	25⁰-20⁰	25⁰-15⁰

Conversely, Table 2 depicts the post-intervention findings, showing a significant improvement in the range of motion for these joints.

**Table 2:** Post-intervention range of motion of affected hand (left hand) MCP - metacarpophalangeal joint; PIP - proximal Interphalangeal joint; DIP - distal Interphalangeal joint; NA - not assessed

Movements		Pre-rehabilitation				
		Thumb	Index Finger	Middle Finger	Ring Finger	Little finger
MCP	Flexion	0⁰-50⁰	5⁰-80⁰	10⁰-75⁰	5⁰-80⁰	5⁰-85⁰
	Extension	50⁰-0⁰	80⁰-5⁰	75⁰-10⁰	80⁰-5⁰	85⁰-5⁰
PIP	Flexion	0⁰-85⁰	5⁰-110⁰	15⁰-105⁰	5⁰-110⁰	5⁰-115⁰
	Extension	85⁰-0⁰	110⁰-5⁰	105⁰-15⁰	110⁰-5⁰	115⁰-5⁰
DIP	Flexion	NA	5⁰-65⁰	20⁰-60⁰	5⁰-65⁰	5⁰-70⁰
	Extension	NA	65⁰-5⁰	60⁰-5⁰	65⁰-5⁰	70⁰-5⁰

Table 3 displays the pre and post-break test results of the left hand (affected hand), it aims to assess the muscle's capability to withstand gradual increments of pressure, probing various facets of neuromuscular control distinct from assessments against fixed resistances with grading conducted according to Kendall muscles testing and function.

**Table 3:** Pre- and post-break test results of the affected hand (left hand) with grading conducted according to Kendall muscle testing and function

Muscles	Pre-rehabilitation	Post-rehabilitation
Flexor digitorum superficialis, flexor digitorum profundus, lumbricals (Patient was asked to make a fist)	Grade 1	Grade 4
Extensor digitorum, extensor indicis, extensor digiti minimi, lumbricals (Patient was asked to open the hand)	Grade 1	Grade 4
Dorsal interossei (Patient was asked to spread fingers apart)	Grade 0	Grade 4
Palmar interossei (Patient was asked to bring fingers together)	Grade 1	Grade 4
Flexor pollicis brevis, opponens pollicis, adductor pollicis, abductor pollicis brevis (Patient was asked to touch thumb to each fingertip)	Grade 3	Grade 5

Radiological investigations

**Figure 1:**
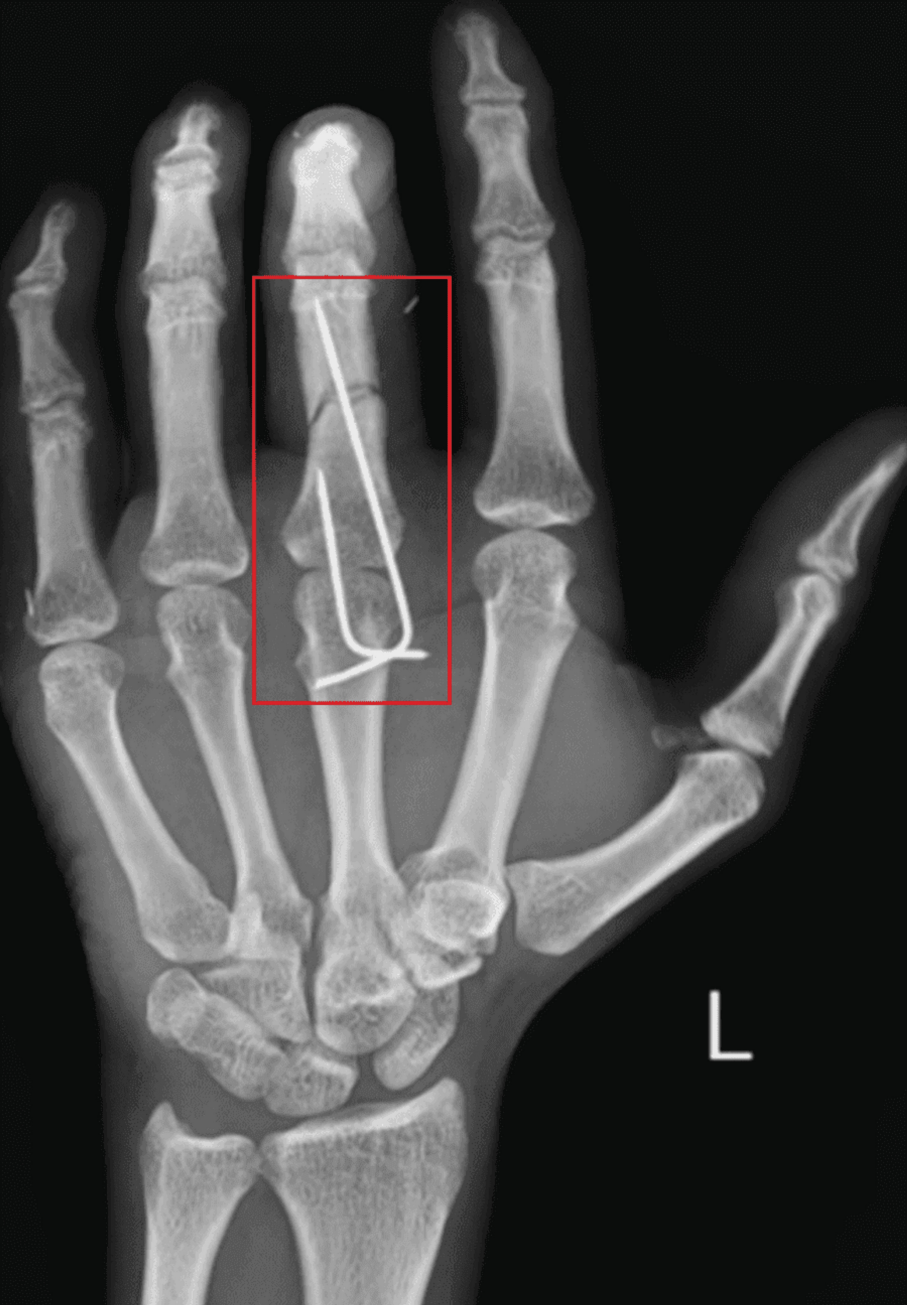
Radiograph of left hand in AP view showing K-wire fixation over displaced fracture of middle phalangeal of third finger under the classification of type 3 phalangeal fracture, resulting from displacement and a lack of direct contact between the bone fragments

Figure 1 depicts radiographs of the left hand in the anterior view.

**Figure 2:**
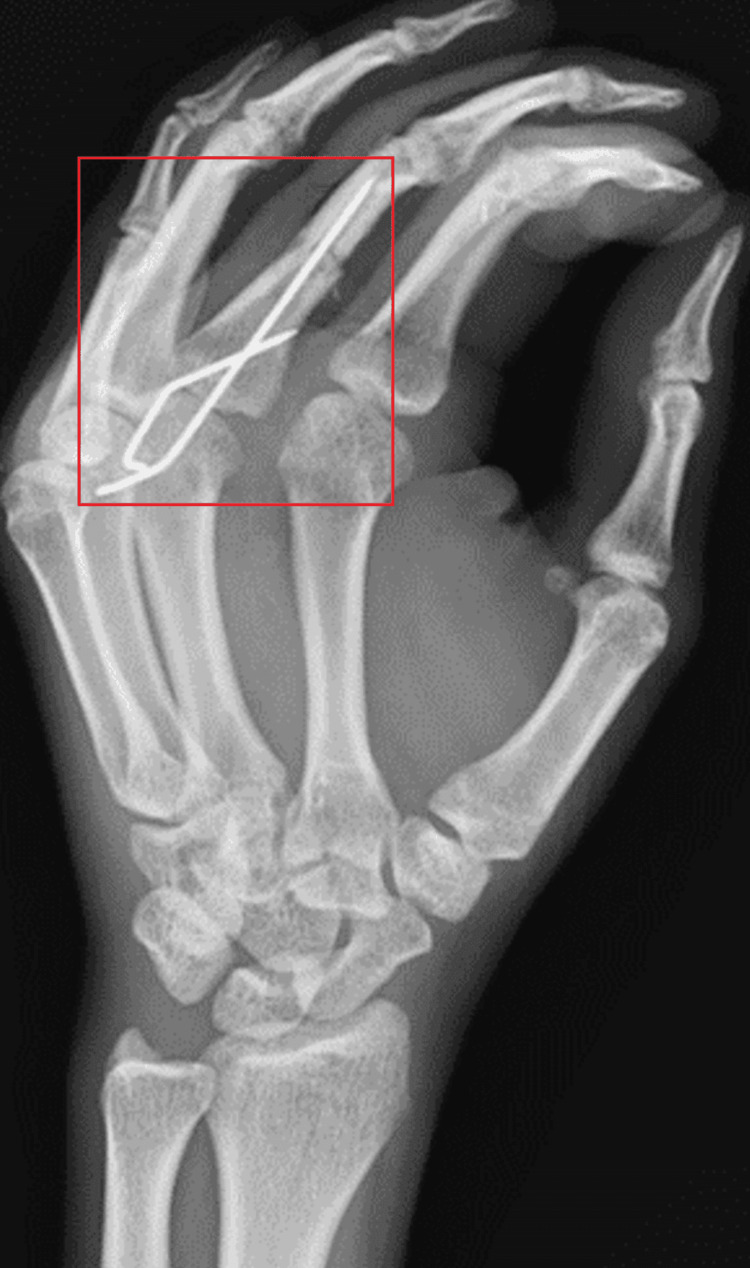
Oblique view of the left hand shows K-wire fixation over displaced fracture of the middle phalangeal of the third finger

Figure 2 shows a radiograph of the left hand in the oblique view.

Table 4 depicts the timeline of events.

**Table 4:** Timeline of events

Timeline	Events
First week of November	The patient had an injury and was diagnosed with a middle phalangeal fracture of the middle finger of the left hand on the same day patient underwent surgery in which K-wire fixation was done with wound suturing over the fracture site
Second week of November	K-wire was removed
Second week of December	The patient was referred to physiotherapy

Physiotherapy management 

Table 5 shows a summary of rehabilitation given to the patient in 4 weeks for 3 days a week.

**Table 5:** Summary of rehabilitation given to the patient in four weeks for three days a week NA - not applicable; MWM - movement with mobilization; PNF - proprioceptive neuromuscular facilitation

Sr No.	Goal	Intervention	Rationale	Dosage
1	Patient education about the condition and possible outcomes of rehabilitation	Patient education about the role of physiotherapy rehabilitation and the condition.	To gain adherence to physiotherapy sessions and to prevent secondary complications	NA
2	To reduce pain	Hydrocollator pack	Help to improve blood circulation, reduce pain, and promote relaxation of the muscles and tissues surrounding the injured area	10 minutes 3 times a week
3	To improve range of motion	Passive range of motion to all the joints of the affected hand	This helps to improve joint flexibility, prevent stiffness, and promote tissue healing by gently stretching the muscles, ligaments, and tendons around the injured phalangeal fracture	10 Reps with 2 sets
4	To restore normal joint function	Mulligan's MWM	To restore normal joint mechanics, improve joint mobility, and reduce pain by applying sustained forces while simultaneously performing specific movements	3 times a week
5	To improve flexibility	Percussion massage gun therapy	It helps to increase blood flow, relax a tight muscle, and improve flexibility	10 min per session 3 times a week
6	To enhance joint stability, proprioception, and motor control	PNF	PNF techniques involve specific patterns of movement combined with resistance to improve joint stability, proprioception, and motor control, it engages the neuromuscular system to enhance coordination and functional movements.	10 Reps with 2 sets
7	To improve grip strength	Squeezing a stress ball hand gripper exercises	Grip strengthening exercises target the muscles of the hand and forearm responsible for gripping and grasping activities. By incorporating resistance training techniques by enhancing the patient's ability to perform activities of daily living and occupational tasks requiring manual dexterity and strength.	10 Reps with 3 sets, rest for 1 min after each set
8	To improve grip strength, dexterity, and coordination	Blocking exercises	To improve grip strength, dexterity, and coordination, essential for the patient's functional recovery and return to daily activities by incorporating the use of external resistance	10 Reps with 2 sets

Follow-up and outcome measures

Pre- and post-physiotherapy rehabilitation outcomes are mentioned in Table 6 and suggest a significant improvement.

**Table 6:** Pre and post-physiotherapy rehabilitation outcomes, which suggest a significant improvement NPRS - Numerical Pain Rating Scale; DASH - disability of the arm, shoulder, and hand

Sr No.	Outcome measures	Pre-treatment	Post-treatment
1	NPRS on movement	8/10	3/10
	NPRS at rest	4/10	1/10
2	DASH	96.4	19.2

## Discussion

The case is presented on the management of rehabilitation of a middle phalangeal fracture, particularly in the context of industrial incidents. Hand injuries in industrial settings are not uncommon and often present unique challenges due to the potential severity of trauma and the necessity for prompt and effective intervention to minimize long-term disability and facilitate return to work [13,14]. In this case, surgical intervention, in the form of K-wire fixation, was employed to stabilize the fracture. K-wire fixation is a commonly used technique for the management of hand fractures due to its simplicity, cost-effectiveness, and accessibility. However, K-wire fixation has challenges, including the risk of postoperative complications [15]. Postoperative rehabilitation plays an important role in restoring hand function, facilitating recovery, and promoting return to work. The rehabilitation protocol approach is aimed at addressing pain, improving range of motion, and enhancing grip strength [16]. Passive range of motion exercises, Mulligan's MWM techniques, and blocking exercises are essential components of the rehabilitation regimen, aiming to prevent stiffness, muscle weakness, and joint adhesions commonly associated with immobilization. Additionally, grip-strengthening exercises are incorporated to enhance hand strength and functionality, which is essential for the patient's ability to perform ADLs and eventually return to work [17]. PNF techniques contribute to enhancing various aspects of physical fitness, including muscular strength, endurance, joint stability, mobility, neuromuscular control, and coordination. These techniques are directed towards enhancing the overall functional capacity of patients [18]. Mulligan's MWM involves blending passive and active mobilization methods and can be utilized to address a variety of musculoskeletal ailments, leading to the restoration of function over both short and long durations. By addressing positional faults of the bony segments and facilitating rapid pain relief and movement restoration, MWM offers a promising approach to rehabilitation following proximal phalangeal fractures [19,20].

## Conclusions

The study has found the effectiveness of early mobilization and targeted exercises in managing middle phalanx fractures treated with K-wire fixation. By implementing a comprehensive rehabilitation protocol focused on pain relief, range of motion improvement, and grip strength enhancement, significant improvements in patient outcomes were achieved, and treatment results in promoting successful return to work, particularly in industrial settings.
